# Oxidative lesions of DNA in membranous glomerulonephritis associated with the infection with hepatitis B virus

**DOI:** 10.1186/1471-2334-14-S7-P51

**Published:** 2014-10-15

**Authors:** Mircea Niculae Penescu, Corina-Daniela Ene (Nicolae), Emanoil Ceauşu, Ilinca Nicolae

**Affiliations:** 1 Carol Davila Clinical Hospital of Nephrology, Bucharest, Romania; 2 Clinical Hospital of Infectious and Tropical Diseases “Dr. Victor Babeş”, Bucharest, Romania

## Background

The kidneys are known as a target of hepatitis viruses. The renal impairment associated with hepatitis B virus (HBV) could be represented by lesions of glomeruli, or by injury of the renal tubules. Membranous nephropathy is the most common form of glomerulonephritis associated with HBV infection. We aimed to develop a prospective study that evaluated the relation between serum levels of 8-hydroxy-2-deoxiguanosine (8OHdG) and total antioxidant status (TAS) in patients with membranous glomerulopathy associated with HBV infection.

## Material and Methods

The study included a group of 18 patients with chronic infection with HBV and membranous nephropathy with nephrotic syndrome, without renal failure (eGFR >60mL/min/1.73sqm), and a group of 20 patients with chronic infection with HBV without membranous glomerulonephritis. The patients did not receive any treatment for the mentioned diseases before the inclusion in the study. The groups were similar for demographic and nutritional status characteristics. The assessment of 8OHdG, a metabolite of oxidative damage of DNA, was made by ELISA method. TAS was determined by spectrophotometric method.

## Results

In patients with chronic HBV infection and membranous nephropathy, 8OHdG (ng/mL serum) had higher levels than in patients with chronic HBV infection without renal disease (14.31±4.73 versus 8.11±2.66, p<0.05). Serum levels of TAS (mmol/L serum) were significantly lower in patients with membranous glomerulopathy compared with those without nephropathy (0.82±0.27, versus 1.01±0.31, p<0.05). A statistically strong negative correlation was determined between 8OHdG and TAS in patients with glomerulopathy associated with HBV infection.

## Conclusion

The negative association between serum levels of 8OHdG and TAS in patients with membranous nephropathy induced by HBV infection suggests an important role of oxidative stress in the development of renal diseases. The adjustment of imbalance between levels of reactive oxygen/nitrogen species and those of antioxidants in the human body could represent a goal for a better management of renal diseases.

